# Triggering Immune System With Nanomaterials for Cancer Immunotherapy

**DOI:** 10.3389/fbioe.2022.878524

**Published:** 2022-04-14

**Authors:** Qiyan Li, Yulin Liu, Zihua Huang, Yajie Guo, Qingjiao Li

**Affiliations:** The Eighth Affiliated Hospital, Sun Yat-sen University, Shenzhen, China

**Keywords:** nanomaterials, cancer immunotherapy, delivery system, photothermal immunotherapy, photodynamic immunotherapy, nanovaccines

## Abstract

Cancer is a major cause of incidence rate and mortality worldwide. In recent years, cancer immunotherapy has made great progress in the preclinical and clinical treatment of advanced malignant tumors. However, cancer patients will have transient cancer suppression reaction and serious immune related adverse reactions when receiving immunotherapy. In recent years, nanoparticle-based immunotherapy, which can accurately deliver immunogens, activate antigen presenting cells (APCs) and effector cells, provides a new insight to solve the above problems. In this review, we discuss the research progress of nanomaterials in immunotherapy including nanoparticle-based delivery systems, nanoparticle-based photothermal and photodynamic immunotherapy, nanovaccines, nanoparticle-based T cell cancer immunotherapy and nanoparticle-based bacteria cancer immunotherapy. We also put forward the current challenges and prospects of immunomodulatory therapy.

## Introduction

### An Overview of Cancer Immunotherapy

Cancer is a major cause of incidence rate and death in the world. In 2020, there were an estimated 19.3 million new cancer cases and 10 million deaths worldwide (Sung et al., 2021). In view of its high risk and mortality, efforts have been made to develop effective treatments to combat cancer (Li Z. et al., 2017). The conventional anti-tumor treatment methods including surgery, radiotherapy, chemotherapy and molecular targeted therapy, have certain disadvantages, such as low response rate, high side effects and high recurrence rate (Gao et al., 2020). In recent years, cancer immunotherapy has achieved significant successes through the enormous number clinical trials approved by the US Food and Drug Administration (FDA). In immunotherapy, the agents are designed to trigger (activate or boost) the immune system to attack cancer cells, which is a natural mechanism in human body. These therapies can provide potent and prolonged anti-cancer responses for a subset of patients who are resistant to conventional therapy, ultimately considering as a promising strategy to cancer.

The main classes of immunotherapies are immune checkpoint inhibitors (ICIs) (Sharma and Allison, 2015; Ribas and Wolchok, 2018), chimeric antigen receptor (CAR) T cell therapies (June et al., 2018; Schultz and Mackall, 2019), lymphocyte-activating cytokines, agonistic antibodies against co-stimulatory receptors, and cancer vaccines (Schumacher et al., 2019). Since the approval of the first immune checkpoint inhibitor, Ipilimumab (CTLA-4), by the FDA in 2011, cancer immunotherapy has experienced rapid development (Rosenberg, 2005). Between 2014 and 2018, the field of immunotherapies has witnessed the approval of eight new anticancer drugs by FDA, including programmed death/ligand 1 (PD-1/PD-L1) blockade and CAR T-cell therapy (Sun et al., 2019). In the past decade, the number of cancer immunotherapy has been massively increased, many of which are in clinical trials. These breakthrough advances in immunotherapy for cancer treatment have been recognized by the world, creating a new milestone in immunotherapy. Despite the great success of immunotherapy, its clinical application in cancer treatment still faces challenges in terms of efficacy and safety.

### An Overview of Nanomedicine

Nanomedicine which is referred to the application of nanotechnology in medicine has become extensively widespread because of its advantages in diagnosis and therapy (Peer et al., 2007). In biology system, nanomedicines are formulation of therapeutics with lipids, polymers or inorganic materials, and can act as carrier of pharmaceuticals to direct the particles to a specific organ or cell type (Fokong et al., 2012; Lammers et al., 2012; Beech et al., 2013; Li et al., 2013; Shin et al., 2013; Woodman et al., 2021). Nanoscale dimension typically refers to particulate described to 1–100 nm in size in drug delivery systems. Therapeutic agents can selectively accumulate in pathological regions and specifically release pharmacological effects in the site, avoid undesired off-target effects, and overcome the danger of severe immune toxicities, which occur in the case of other systemic administration (Peer et al., 2007; Morachis et al., 2012).

Tumor targeting by nanomedicines is typically mediated via two main mechanisms, including passive targeting and active targeting (Matsumura and Maeda, 1986; Mi et al., 2020). The uncover of mechanism for passive targeting dates back to the year 1986, when Matsumura and Maeda (Matsumura and Maeda, 1986) started to reveal Enhanced Permeability, as well as Jain and colleagues (Gerlowski and Jain, 1986) started to explore Retention (EPR) effect. Active targeting relies on the functionalized nanoparticles (NPs) with targeting molecules, such as antibodies or peptides, which can increase internalization of drugs at the pathological site. In this case, drug-to-antibody ratios can greatly exceed the ratio of conventional antibody-drug conjugates (Kulkarni et al., 2018). Discuss include the fact recently indicates that both strategies have difference in several perspectives, including overall targeting efficiency, specific cell delivery, formulation complexity and translational potential (Lammers et al., 2012; Lammers et al., 2016).

The field has witnessed the success of approximately 50 nanomedicine therapies approved by the FDA for cancer and other diseases (Bobo et al., 2016). Among the main future ways forward is the combination of nanomedicines with immunotherapy, a therapeutic strategy that has been extensively studied preclinically (Wang et al., 2018; Saeed et al., 2019; Sang et al., 2019; Sun et al., 2019) and is also already being explored in the clinic (Yu et al., 2021).

### Opportunities of Nanomedicine in Immunotherapy

Although immunotherapy has achieved excellent results in clinical practice, there are still many problems that need to be solved. In terms of safety, immunotherapy can induce transient suppression of cancer response and severe immune-related adverse effects in patients treated with immune checkpoint inhibitors (Bowyer et al., 2016; Milling et al., 2017). As observed with chimeric antigen receptor-T cell therapy, the immune status of patients has been significantly improved. However, the large number of injected immune cells affect the homeostasis of the immune environment and overproduce cytokines, which trigger cytokine storm (Predina et al., 2013). In addition, drugs of immunotherapy are usually administered clinically by intravenous injection. This approach shortens the time of drug delivery to the tumor tissue but loses the precise control of drug distribution *in vivo*. As a result, utilization of the drug is significantly reduced, and meanwhile it may cause systemic random activation of the immune system and induce severe immunotoxicity (Predina et al., 2013).

The emergence of nanocarrier-based delivery systems has provided new perspectives for cancer immunotherapy. The loading of immune agents into nanocarriers, considering its promising biocompatibility and stability, can effectively improve the solubility and bioavailability of hydrophobic drugs, prolong the time of drug circulation in the body, and avoid the recognition and clearance by the immune system. It also significantly increases the utilization of immune agents in the body, achieves precise targeting of immune cells or tumor tissues, and reduces toxic side effects, etc., (Gao et al., 2019; Martin et al., 2020). Meanwhile, through the surface modification of target molecules or enhancing EPR, nanocarriers can effectively deliver drugs, antibodies, immunodulators or functional molecules to tumor tissues (Sindhwani et al., 2020). This method can achieve enrichment of drugs and antibodies, regulation of local immunity, improvement of immunosuppressive microenvironment, enhancing the effect of tumor immunotherapy (Sindhwani et al., 2020).

## NP-Based Delivery Systems for Cancer Immunotherapy

**FIGURE 1:**
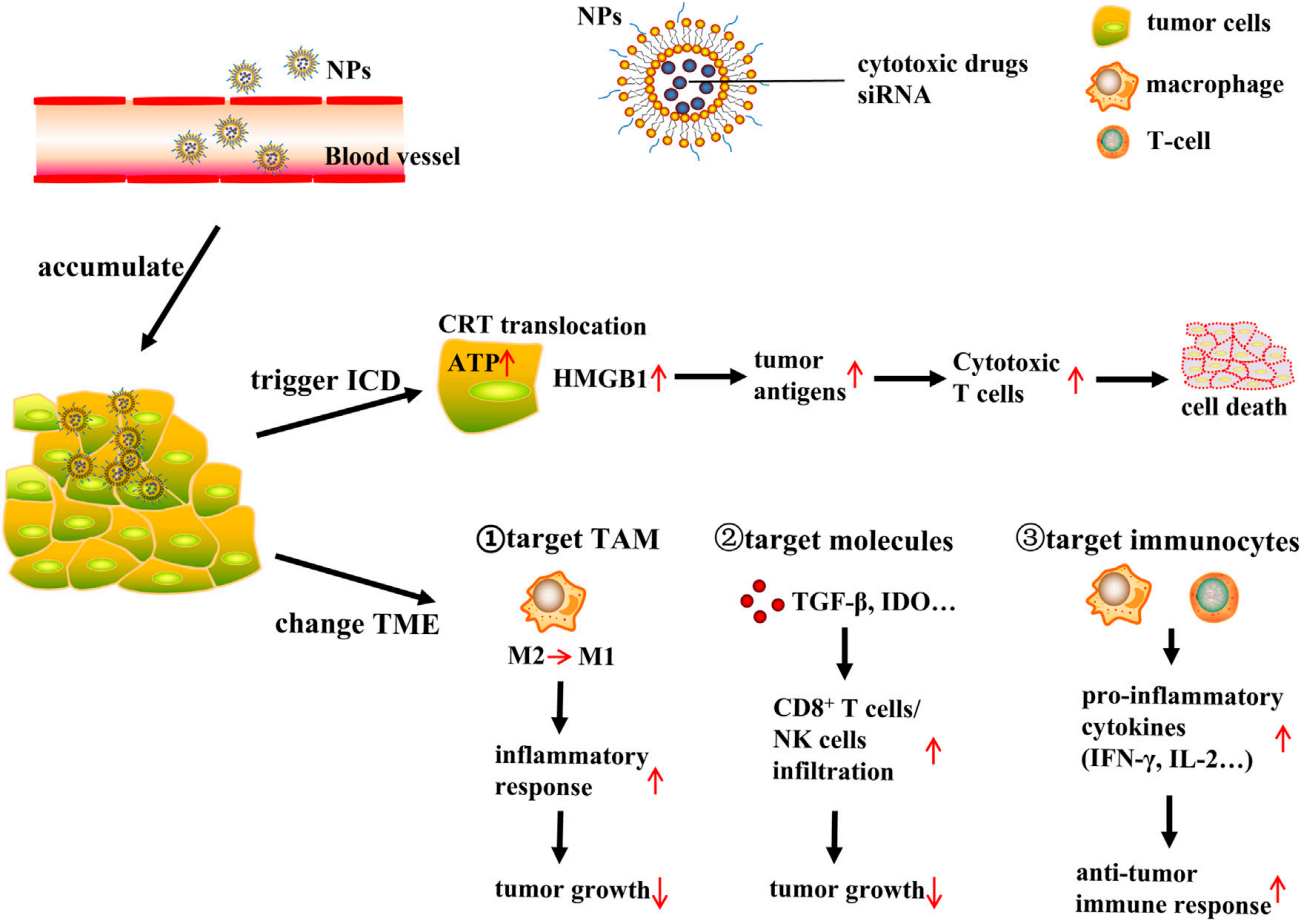
NP-based delivery systems for cancer immunotherapy. NP (nanoparticles); ICD (immunogenic cell death); CRT (calreticulin); ATP (adenosine triphosphate); HMGB1 (high mobility group protein B1); TME (tumor microenvironment); TAM (tumor-associated macrophages). NP-based tumor targeted delivery systems deliver ICD inducing cytotoxic drugs or siRNA to tumor site, then NPs accumulate in the tumor interstitial space, further trigger ICD or change TME to induce immune response and finally lead to tumor cells death. NPs release ICD inducing cytotoxic drugs, inducing CRT translocate to cell surface, ATP release, and HMGB1 expression increase. This triggers tumor antigens process and produce cytotoxic T cells to induce tumor cells death. On the other hand, NPs delivery system could change TME through targeting TAM, immunosuppressive molecules and immunocytes respectively. Target TAM: NPs deliver drugs or siRNA to tumor cells, polarizing macrophages from M2 phenotype to M1 phenotype, and then stimulating inflammatory response, finally suppressing tumor growth. Target immunosuppressive molecules: NPs could act on TME related immunosuppressive molecules such as TGF-β and IDO, this will increase infiltration of CD8^+^ T cells and NK cells, resulting in tumor growth inhibition. Target immunocytes: NPs could directly act on immune cells such as macrophages and cytotoxic T cells and these cells will produce pro-inflammatory cytokines including IFN-γ and IL-2, leading to antitumor immune response increase to suppress tumor growth.

An increasing number of nanomedicine agents have been used synergistically with immunotherapeutic agents to complement the previously singular cancer therapeutic approaches. The impact of nanoparticle-based delivery systems on tumor immunity is currently being explored (Figure 1).

### NPs Delivery System of Immunogenic Cell Death Inducing Cytotoxic Drugs

It is reported that low doses of chemotherapeutic agents can activate apoptotic pathways and trigger immunogenic cell death (ICD), which is a specific mode of cell death, as well as an important trigger and enhancer of anticancer immunity (Gupta et al., 2021). ICD in tumors induced with cytotoxic drugs produces a large number of tumor-associated antigens *in situ*, which active the immune system against tumors, conducting to a safe and efficient novel tumor immunotherapy (Bracci et al., 2014; Brown et al., 2018). ICD inducers identified in current studies include doxorubicin, 5-fluorouracil, gemcitabine, paclitaxel, mitoxantrone, and oxaliplatin (Bezu et al., 2015).

Classical features of ICD are damage-associated molecular patterns (DAMPs), including the translocation of calreticulin (CRT) to the cell surface, the release of adenosine triphosphate (ATP), and overexpression of high mobility group protein B1 (HMGB1) into the extracellular environment (Krysko et al., 2012; Kadiyala et al., 2019). Alerted by these features, natural antigen presenting cells (APCs) in the immune system ingest and process tumor antigens, and produce cytotoxic T cells, and then migrate to eradicate tumors and metastases. Therefore, the efficacy of immune checkpoint blockade therapies can be effectively improved by potentiating the ICD (Binnewies et al., 2018).

In general, anticancer chemotherapy frequently entails the challenge of resulting in high off-target toxicity in normal and immune cells due to its ability to simultaneously kill malignant cancer cells and other normal cells. As a result, unwanted systemic toxicity and immunosuppression can be observed in patients who have given combination therapy with cytotoxic drugs. The NP-based tumor-targeted delivery systems deliver macromolecules including NPs and nanocomplexes into the tumor interstitial space to accumulate within the tumor tissue, which effectively increases the duration of drug’s retention at the tumor site and boosts the EPR effect, yielding a maximum therapeutic effect with low toxicity (Maeda et al., 2000; Gu et al., 2018; Pusuluri et al., 2019). On the other hand, the rapid and enhanced cellular uptake of NPs enhances the tumor-specific immune response (Dai et al., 2017; Golombek et al., 2018). The NP-based drug delivery system for ICD-induced anticancer drugs achieves tumor-specific delivery of cytotoxic agents and suppresses immunosuppression in tumor tissues (Zhang B. et al., 2017).

Recent studies also show that ICD-inducing nanomedicines can be combined with immunotherapy to enhance ICD to potentiate the cancer-immunity cycle (Kuai et al., 2018). For example, Rios-Doria’s group recently showed that the combination therapy consisting of doxorubicin-loaded liposomes (Caelyx/Doxil®) and other clinically relevant immunotherapeutics substantially enhanced antitumor immune response by promoting (*via* ICD) the proliferation of dendritic cells (DCs) and CD8^+^ T cells. Frequently used immunotherapeutic agents include anti-PD-1, -PD-L1 and -CTLA4 antibodies, as well as tumor necrosis factor receptor alpha agonists. It is reported that Doxil was less potent in immunodeficient mice than in immunocompetent mice, and the experiment *in vivo* provided preliminary evidence of the immune potentiation effects of Doxil (Rios-Doria et al., 2015). Zhao et al. encapsulated ICD-inducing agent oxaliplatin (OXA) into nanoparticles. Compared with free OXA treatment, tumor cells treated with OXA-loaded nanoparticles released more DAMPs, inducing more antitumor immune responses produced by DC and T lymphocytes (Zhao et al., 2016). An exemplary study in this regard was published by Zhang et al., who combined doxorubicin, matrix metalloproteinase (MMP)-cleavable peptide and hyaluronic acid (HA) to a nano-sized prodrugs. The nanomedicines intensified antitumor immune response by upregulating interferon-γ (IFN-γ) and PD-L1 (Gao et al., 2018). Similar beneficial effects were reported by Zan et al. as well, who encapsulated a new type of ICD-inducing agent into nanoparticles. The nanoinducer was generated by packing curcumin (CUR) and iron oxide nanoparticles (IONPs) into disulfide-bond-incorporated dendritic mesoporous organosilica nanoparticles (DDMON) and abbreviated as DDMON-CUR-IONP. DDMON-CUR-IONP significantly amplified the oxidative stress pharmacological properties in the tumor immune microenvironment through complementary pharmacological activities, eventually triggering an effective systemic immune response and ICD. It avoids serious adverse effects compared to conventional nanoinducer (Dai et al., 2020). These improvements stemmed from that the NP-based drug delivery system can more effectively target chemotherapeutic agents to cancer tissues and overcome the lymphotoxicity effectively induced by free chemotherapeutic agents. It is anticipated that in the next couple of years, researchers will synthesize a significant number of novel nanopharmaceutical agents specifically designed to induce ICD in tumor cells without toxic effects on normal cells.

### NPs Delivery System for Tumor Immune Microenvironment

The tumor microenvironment (TME) is a heterogeneous environment composed of extracellular matrix (ECM), tumor-infiltrating lymphocytes, cancer-associated fibroblasts (CAF), myeloid suppressor cells, regulatory T cells, and tumor-associated macrophages (TAM). In addition, the TME is filled with soluble proteins such as transforming growth factor beta (TGF-β), cyclooxygenase 2 (COX-2) and epidermal growth factor (EGF) (Lindau et al., 2013; Emon et al., 2018). The synergistic relationship of these components leaves the TME in an immunosuppressed state, consequently supporting the development, progression and metastasis of tumor and limiting the function of APCs and T cells (Joyce and Fearon, 2015). The utilization of nanomedicines that modulate the TME is another important strategy of promoting the efficacy of anticancer immunotherapy, especially for the “cold” solid tumor with low immunogenicity.

### NPs Delivery System Targeting Tumor-Associated Macrophages

Tumor-Associated Macrophages (TAMs) are major population of immunomodulatory cells in tumors, accounting for approximately 50% of solid tumor tissues. TAM are implicated in regulating immune processes in the TME by releasing immunosuppressive cytokines, participating in tumor angiogenesis, inhibiting proliferation and activation of T cells, as well as promoting tumor cell growth, invasion and metastasis (Franklin et al., 2014). During the process from the early stage of tumor formation to tumor metastasis, TAM with M2-like phenotype (M2) typically take the dominant position to enhance the invasive and metastatic ability of tumor cells and promote the stocking and continuous growth of tumor cells, and ultimately achieve the purpose of suppressing anti-tumor immune activity (Ruffell et al., 2012; Colegio et al., 2014). In addition, TAM are generally dominated by cells with an M2-like phenotype associated with Th2 immune response, whereas the M2-like phenotype can still be repolarized into the M1-like phenotype which promotes inflammatory response and suppresses neoplasia. Accumulating evidence has confirmed that the potential of macrophage recruitment chemokines (CCL2, CCL3, CCL4, and CCL5), CSF-1 and VEGF as a clinical therapeutic target to prevent malignancy progression by interrupting the recruitment of TAMs (Halama et al., 2016; Argyle and Kitamura, 2018). To inhibit CCL2 and its cognate receptor CCR2 axis, Shen et al. synthesized siCCR2-encapsulated cationic nanoparticle (CNP/siCCR2) to inhibit primary tumor progression and further metastasis by reducing the abundance of TAMs and altering the immunosuppressive tumor microenvironment (Shen et al., 2018). In another recent study, Trac et al. designed KLAK-MCP-1 micelles, consisting of a CCR2-targeting peptide sequence and apoptotic KLAK peptide, which were effective in inhibiting tumor growth by blocking infiltration of TAMs in a subcutaneous B16F10 murine melanoma model (Trac et al., 2021). Zhang et al. loaded gemcitabine onto ultrasmall copper nanoparticles (Cu@CuO x) for PET-guided drug delivery, which could specifically target CCR2 and synergize the therapeutic effects of gemcitabine, ultimately slowing the growth of pancreatic ductal carcinoma (Zhang X. et al., 2021). Analogously, Chen et al. developed a nano-spray gel, and achieved effective clearance of residual carcinoma cells after tumor surgery via polarizing macrophages towards an M1 phenotype (Chen et al., 2019). In this regard, targeting TAM in the TME is a promising strategy for improving cancer therapy.

### NPs Delivery System Targeting Immunosuppressive Molecules

Nanomedicines can also act on molecules in the TME, such as TGF-β and Indoleamine 2,3-dioxygenase (IDO), which have immunosuppressive effects. TGF-β is an important immunosuppressive factor in tumors, which contributes to the formation of promotion of tumor inflammatory microenvironment, and diminishes the efficacy of checkpoint inhibition immunotherapy (Mariathasan et al., 2018; Tauriello et al., 2018). To address the drawbacks of TGF-β inhibitors in clinical trials with poor pharmacokinetic behavior and high systemic toxicity with insufficient tumor permeability, Park and co-workers developed a novel nanoparticle for the co-delivery of TGF-β inhibitor and interleukin-2. They revealed the significant increase in infiltration of CD8^+^ T cells and NK cells, resulting in the significant inhibition of tumor growth and alleviation of immunosuppression (Park et al., 2012). Huang et al. developed a TGF-β siRNA-containing nanoformulation that synergized with cancer vaccination, which achieved silence of TGF-β expression, to significantly enhance its therapeutic effect on advanced tumors (Xu et al., 2014). As one of the key factors contributing to tumor immune tolerance, IDO promotes the conversion of tryptophan to kynurenine, while the former one is essential for T cell proliferation and killing and the latter one is a potent T cell suppressing metabolite (Yuan et al., 2017). A variety of small molecule IDO inhibitors have been extensively applied in (pre-)clinical trials for adjuvant cancer immunotherapy to improve outcomes of immunotherapeutic interventions. Recently, Lu and colleagues combined an IDO inhibitor indocimod with the ICD inducer OXA in lipid-coated mesoporous silica nanoparticles, achieving the inhibition of tumor growth in a mouse model of pancreatic ductal adenocarcinoma and significantly prolonging the survival period of mice with tumors (Lu et al., 2017). Han et al. developed a nanoparticle NLG-RGD NI, consisting of a peptide backbone and a targeting motif, as a carrier for the IDO inhibitor NLG919. After NLG-RGD NI was targeted to tumor tissue and taken up, it consistently inhibited IDO activity and reduced systemic toxicity caused by the non-specific distribution of NLG919 (Han et al., 2020).

### NPs Delivery System Targeting Immunocytes

In addition, nanomedicines can also be used to influence the function of immune cells directly and positively such as macrophages and cytotoxic T cells in TME. To improve selective and immune-mediated eradication of cancer cells, Yuan et al. constructed a multivalent bi-specific nanobioconjugate engager (mBiNE), which could target HER2 expressed by cancer cells and pro-phagocytosis signals at the same time. In mice implanted HER2high E0771/E2 tumors treated with mBiNE, they found that the infiltration of macrophages and T cells, production of pro-inflammatory cytokines such as IFN-γ and IL-2 were substantially increased. Ultimately mBiNE elicited systemic, durable antitumour immune responses by promoting targeted phagocytosis of tumors by macrophages and enhancing T cells activation (Yuan et al., 2017). Recently, Shae et al. designed stimulator of interferon genes (STING)-activating nanoparticles (STING-NPs) that effectively encapsulated 2’3’ cyclic guanosine monophosphate-adenosine monophosphate (cGAMP), and acted as a delivery of cGAMP. Through triggering innate immune response derived by IFN-I, cGAMP could inhibit tumor growth, enhance tumor immunogenicity, and increase rates of long-term survival of patients. Treatment with STING-NPs triggers systemic antitumor immunity and enhances the efficacy of checkpoint blockade therapies (Shae et al., 2019). Li et al. constructed a novel cancer-derived magnetosome with Fe_3_O_4_ magnetic nanoclusters (MNCs) as the core, wrapped around anti-CD205-modified cancer cell membranes. Magnetic resonance imaging (MRI) enabled the magnetic nanoclusters to remain in the lymph nodes, inducing massive proliferation of cytotoxic T cells and triggering antitumor immunity (Li F. et al., 2019).

In conclusion, NP-based delivery systems can generally enhance anticancer immunity by modulating TME in two different approaches, i.e., by alleviating immunosuppression or by promoting immune activation. Depending on the target site, the delivery system will operate with different strategies to enhance the effect of tumor immunity.

## NP-Based Photothermal and Photodynamic Immunotherapy

**FIGURE 2:**
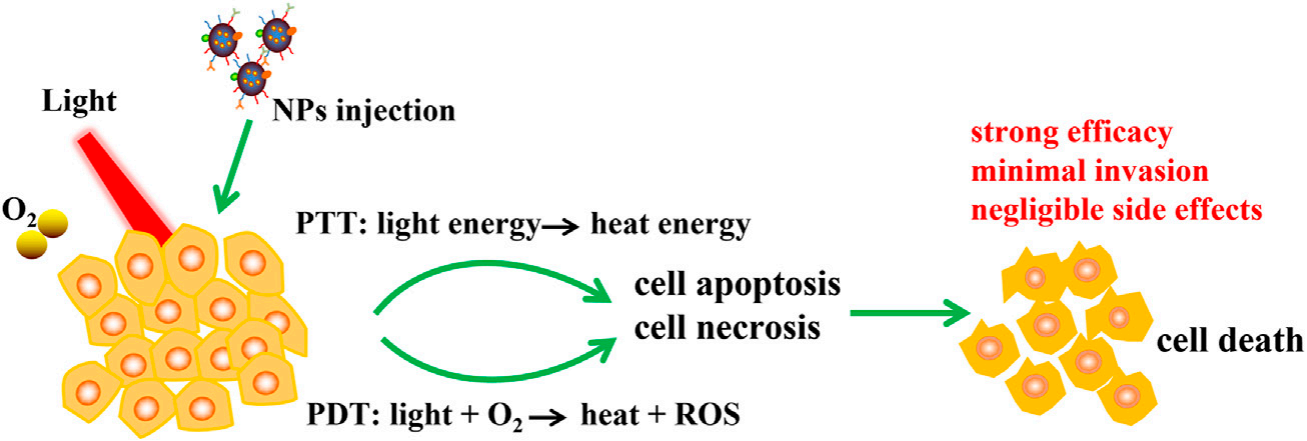
Schematic illustration of NPs based PTT and PDT therapy. NPs was injected to tumor, PTT and PDT convert light energy into heat energy and induce ROS with the help of excitation light and oxygen, then cell apoptosis and necrosis were occurred and finally leading to cell death. PTT and PDT have advantages including strong efficacy, minimal invasion and negligible side effects.

In recent years, phototherapy based on nanoparticles, such as photothermal therapy (PTT) and photodynamic therapy (PDT), has attracted extensive attention for tumor treatment because of its strong efficacy, minimal invasion and negligible side effects. PTT and PDT can not only kill tumor cells directly through heat and reactive oxygen species (ROS), but also induce a variety of antitumor effects. In particular, PTT and PDT lead to a large number of tumor cell deaths and trigger immune responses, including redistribution and activation of immune effector cells, expression and secretion of cytokines, and transformation of memory T lymphocytes (Figure 2).

### NP-Based Photothermal Therapy With Immunotherapy

**FIGURE 3:**
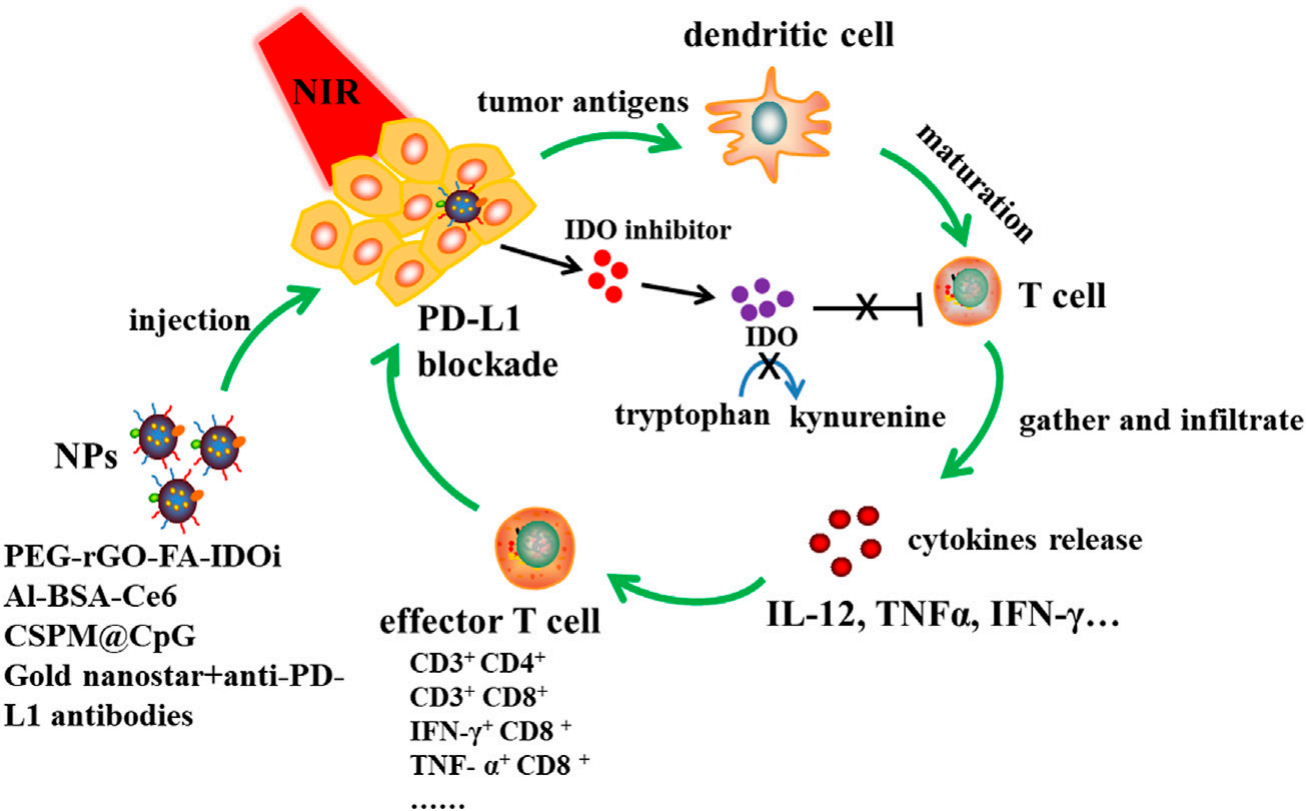
NPs based PTT immunotherapy together with IDO inhibition and PD-L1 blockade. Designed NPs such as PEG-rGO-FA-IDOi, Al-BSA-Ce6, CSPM@CpG, Gold nanostar-anti-PD-L1 antibodies were injected to tumor site, and then immune responses were triggered at a specific light wavelength. Tumor antigens are produced, dendritic cells are maturated into T cells, then T cells gather and infiltrate tumor site, promoting cytokines release like IL-12, TNFα and IFN-γ to increase effctor T cells proportion. NPs release IDO inhibitor, which could inhibit IDO converting tryptophan to kynurenine. This was beneficial to T cell proliferation. What’s more, NPs combined with anti-PD-L1 antibodies could induce PD-L1 blockade and then enhance immune response to kill tumor cells.

The application principle of PTT is to use the performance of photothermal conversion nanomaterials to convert light energy into heat energy at a specific light wavelength, thereby killing cancer cells (Figure 3). Compared with the existing tumor treatment methods, PTT has the following advantages: 1) Near-infrared (NIR) can penetrate deeper tissues with minimal damage to healthy tissues (Saneja et al., 2018). 2) In addition to superficial tumors, PTT combined with interventional technology has the potential to treat deep tumors (Hu et al., 2017). 3) Targeted molecular modified nanoparticles are used to drug or nucleic acid delivery, and tumor-specific killing can be achieved after intravenous injection (Zhu H. et al., 2017; Zhu X. et al., 2017). At present, PTT has attracted increasing attention as a non-invasive treatment.

Yan et al. achieved a combination of PTT and PD-L1 blockade by utilizing polyethylene glycol (PEG) and FA functionalized reduced graphene oxide (rGO) -based nanosheets loaded with IDO inhibitor (IDOi) (Yan et al., 2019). Cancer cells could be destroyed significantly by the hyperthermia produced by PTT. After treating with PEG-rGO-FA-IDOi nanosheets, CD45^+^ leukocytes, CD3^+^ CD4^+^ T cells and CD3^+^ CD8^+^ T cells in the distant tumor would increase. Zhu et al. prepared bovine serum albumin nanosystems (Al-BSA-Ce6 NPs) by albumin-based biomineralization with photosensitizer E6 (Ce6) chloride and immune adjuvant aluminum hydroxide (Zhu et al., 2020). When injected intravenously, nanoparticles not only effectively destroyed tumor cells, but also protected animals from tumor re-invasion and metastasis by strongly inducing an anti-tumor immune response. After photoablation, Al-BSA - Ce6 NPs triggered a systemic anti-tumor immune response that T cells gathered in lymph nodes and infiltrated the tumor sites, increasing the levels of serum antibodies and cytokines, and the proportion of IFN-γ^+^ CD8^+^ T cells, TNF- α^+^ CD8^+^ T cells and IFN- γ^+^ CD4^+^ Th1 cells. Zhou et al. constructed a multifunctional tumor treatment platform by successively coating the surface of Cu_9_S_5_ nanocrystals with mesoporous silica shell and manganese dioxide shell, and then adsorbing immune adjuvant (CpG) for synergistic phototherapy and immunotherapy (Zhou et al., 2020a). Under 650 nm laser irradiation and 808 nm NIR laser irradiation, CSPM@CpG nanocomposites could effectively produce ROS and a large amount of heat, leading to cancer cell deaths. In addition, it was found that the nanocomposites can promote the uptake of CpG, and promote the generation of IL-12, TNF- α and IFN- γ. Liu et al. revealed a synergistic immuno-PTT combined therapy strategy (SYMPHONY) that completely eliminated primary tumor and distant untreated tumors in mice carrying MB49 bladder cancer cells, which combined immunosuppression point suppression, anti PD — L1 antibodies and gold nanostar (Liu et al., 2017). Gold nanostar provided mild hyperthermia under NIR radiation, which triggered local and systemic immune responses. Wang et al. combined indocyanine green (ICG)–loaded magnetic silica NIR sensitive nanoparticles (NSNP) to develop temperature activated engineered neutrophils (NE) (Wang et al., 2021). The combination of neutrophil targeting and magnetic targeting increased the accumulation of photothermal agent (PTA) in tumor sites. Under NIR irradiation, NSNP can cause local temperature rise and NE thermal stimulation at the tumor site. High temperature can directly kill tumor cells and also lead to the death of neutrophils. In the case of neutrophil death, it will release active substances with tumor killing effect and kill residual tumor cells, so as to reduce tumor recurrence. Moreover, the composite can significantly enhance the killing effect of photothermal therapy and has no recurrence in animal models with pancreatic tumors.

Heat shock proteins (HSPs) are protective proteins which will be over expressed when cells are stressed by heat, ischemia, heavy metals and toxins. They can repair protein damage and prevent cell apoptosis (Horowitz and Robinson, 2007; Calderwood and Gong, 2016). HSP70 and HSP90 are members of HSP family, participating in the folding and function of a variety of proteins. By regulating the expression of carcinogenic client proteins such as Ras, p53 and Akt, HSP70 and HSP90 are very important for the survival of tumor cells (Cavanaugh et al., 2015). In addition, due to the tolerance of tumor cells to heat stress, overexpression of HSP at tumor sites would lead to low efficiency of PTT (Chen et al., 2017). Therefore, reducing HSP70 and HSP90 in tumor cells not only promotes apoptosis, but also improves the thermal sensitivity of tumor cells (Jego et al., 2013; Ali et al., 2016; Lin et al., 2016). Tang et al. constructed a treatment system (MPEG-AuNR@VER-M) composed of methoxy-polyethylene-glycol-coated-gold-nanorods (MPEG-AuNR) and ver-155008 micelles (VER-M). VER-M promoted tumor cell apoptosis by specifically reducing the expression of HSP70 and HSP90 (Chatterjee et al., 2013; Li H. et al., 2017; Zhang H. et al., 2017; Fan et al., 2017). It is found that *in vitro* study increasing the concentration of VER-M and elevating the temperature of MPEG-AuNR@VER-M can improve the effect of growth inhibitors on human colon cancer (HCT116) cells (Tang et al., 2018). Yang et al. designed a simple strategy to prepare PEG-modified one-dimensional nano coordination polymer (1D-NCPs), and then loaded with gambogic acid (GA), a natural inhibitor of HSP90, which could effectively induce tumor cell apoptosis and achieve low-temperature PTT under mild near-infrared trigger heating (Yang Y. et al., 2017). Wu et al. designed a hollow mesoporous organosilicon nanocapsule (HMONs) nano platform which had an excellent tumor destruction effect after loading indocyanine green (ICG), HSP90 inhibitor and 17AAG were modified with polyethylene glycol (NH2-PEG) (Wu et al., 2018). Zhong et al. designed QE-PEG-Ag_2_S by self-assembly of hydrophobic Ag_2_S nanodots (Ag_2_S NDs), amphiphilic pH-reactive PEG_5k_-PAE_10k_ polymer, and an HSP70 inhibitor quercetin (QE) (Zhong et al., 2020). QE-PEG-Ag_2_S achieved complete tumor ablation without recurrence when irradiated with NIR light for 10 min. It provides a new way for the therapeutic application of Ag_2_S NDs. Wu et al. designed nano catalyst (G/A@CaCO3-PEG) which is composed of calcium carbonate (CaCO_3_) — supported glucose oxidase (GOD) and 2D antimonene quantum dots (AQDS), and further surface modified by lipid bilayer and PEG (Wu et al., 2022). The integrated GOD effectively catalyzes the consumption of glucose, thereby reducing the supply of ATP and subsequently downregulating the expression of HSP. Under the irradiation of NIR light, this effect enhances the efficacy of photothermal hyperthermia induced by 2D AQDS by reversing the heat resistance of cancer cells.

Small interfering RNA (siRNA), as an effective carrier of RNA interference, suppressed the heat shock response and made cancer cells more sensitive to PTT by inhibiting the expression of specific genes and silencing the expression of heat shock proteins. Liu et al. successfully synthesized flower-like gold nanoparticles (GNFs) using CTAC as a soft template, and then loaded siRNA, which was called GNFs-siRNA (Liu Y. et al., 2019). *In vivo* studies showed that due to the synergistic effect of GNF mediated PTT and siRNA triggered inhibition of heat shock response, GNFs-siRNA showed a significant anti-tumor effect in the irradiated HepG2 tumor model. Gold nanorods (GNRs)-siRNA platform with gene silencing ability was fabricated by Wang et al. to improve the efficiency of PTT (Wang et al., 2016). After surface modification, GNRs could deliver siRNA oligomers targeting BAG3, a gene that effectively blocked heat shock response. *In vitro* and *in vivo* experiments showed that GNRs-siRNA nanocomposites increased and promoted apoptosis by down-regulating BAG3 expression under moderate laser irradiation, making cancer cells sensitive to PTT. A folate (FA)-modified polydopamine (PDA) nanodrug for photothermal therapy was designed by Zhang et al. for siRNA delivery to knock down the ROC1 oncogene (Zhang Z. et al., 2021). *In vitro* and *in vivo* experiments show that gene nano drugs combined with PTT can effectively inhibit the proliferation and promote the apoptosis of liver cancer cells. Liu et al. developed a novel multifunctional nanostructure GAL-GNR-siGPC-3, which uses galactose (GAL) as the targeting part of hepatocellular carcinoma (HCC) and gold nanorods (GNR) as the framework to destroy tumor cells under laser irradiation, and the siRNA of Glypican-3 (siGPC-3) which induces specific GPC-3 gene silencing in HCC (Liu Y. et al., 2021). GAL and siGPC-3 can induce targeted silencing of GPC-3 gene in hepatoma cells. The results *in vivo* and *in vitro* showed that GAL-GNR-siGPC-3 could significantly induce the downregulation of GPC-3 gene and inhibit the progression of HCC.

### NP-Based Photodynamic Therapy With Immunotherapy

**FIGURE 4:**
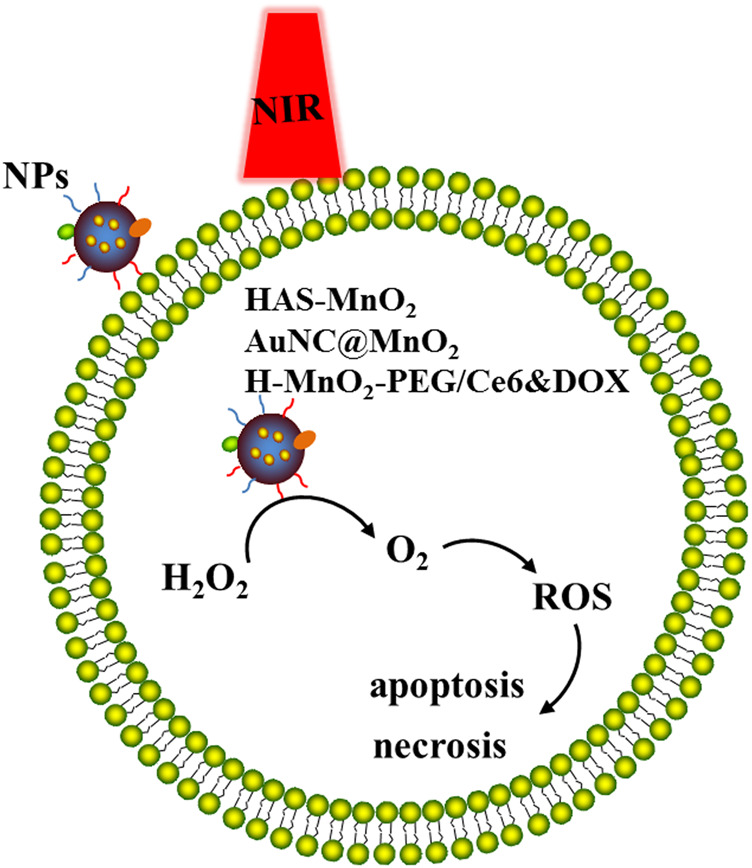
NPs based PDT immunotherapy. In the presence of specific wavelength excitation light, designed nanoparticles like HAS-MnO_2_, AuNC@MnO_2_ and H-MnO_2-_PEG/Ce6&DOX transport oxygen into tumor or *in situ* generation of O_2_ inside the tumor from endogenous H_2_O_2_ with catalysts, which improves tumor oxygenation level and changes tumor microenvironment, increases ROS levels, leading to tumor cell apoptosis and necrosis.

The anti-tumor principle of PDT is that in the presence of molecular oxygen and activated by specific wavelength excitation light, photosensitizers (PSs) are selectively retained in tumor tissues to produce singlet oxygen and other ROS, leading to tumor cell apoptosis and necrosis (Zhang et al., 2018). PDT has the advantages of small trauma, low toxic and side effects, good selectivity and good reproducibility (Park et al., 2017). It is an effective method for clinical treatment of various diseases (Figure 4).

Wu et al. developed an immunotherapy method composed of natural immune activator Astragaloside III (AS) and photodynamic therapy (PDT) reagent chloroe6 (Ce6) [(As + Ce6) @ MSNs-PEG] for colon cancer (Wu et al., 2021). The results showed that *in vitro* (As + Ce6) @ MSNs-PEG could effectively activate NK cells and inhibit the proliferation of tumor cells. Furthermore, it can effectively reach the tumor site *in vivo*, induce immune cells to infiltrate into the tumor, and enhance the cytotoxicity of natural killer cells and CD8^+^ T cells. In Liu’s recent work, they designed a nanocomposite called CE6/MLT@SAB, after light treatment, and the ability of CE6/MLT@SAB-treated cells to activate DCs were significantly increased (Liu H. et al., 2019). Xing et al. constructed a multifunctional nano platform reasonably by pre saturating oxygen fluorinated polymer nanoparticles and encapsulating PS (Ce6) and indoleamine 2,3-dioxygenase (IDO) inhibitor (NLG919) (Xing et al., 2019). The combination of PDT and NLG919 can enhance IFN- γ positive CD8^+^ T cells significantly, producing effective synergistic antitumor immunity. Liang et al. developed a AuNC@MnO_2_ (AM) nanoparticles for oxygen enhanced PDT combined with immunotherapy in the treatment of metastatic triple-negative breast cancer (mTNBC) (Liang et al., 2018). The oxygen enhanced PDT effect of AM can not only effectively destroy the primary tumor, but also induce immunogenic cell death (ICD), release damage related molecular patterns (DAMPs), and then induce DC maturation and effector cell activation, thus effectively stimulating the systematic antitumor immune response against mTNBC.

Tumors are usually exposed to hypoxia. After PDT damages blood vessels, it will cut off the blood supply, further worsen the hypoxic environment, and seriously affect the therapeutic effect, especially in deep tumors (Ma et al., 2019; Zhang Y. et al., 2020). Therefore, it is very important to slow the rapid deterioration of hypoxic environment. pH/H_2_O_2_ dual-responsive nanoparticles were designed by Chen et al. using albumin-coated MnO_2_ (Chen et al., 2016). When MnO_2_ penetrated the tumor, it reacted with H_2_O_2_ and H^+^ to produce oxygen. By alleviating hypoxia, the effect of PDT was enhanced. Yang et al. synthesized MnO_2_ nano-platform which can regulate the hypoxic TME to and enhance the therapeutic effect of PDT (Yang G. et al., 2017). A carbon nitride (C_3_N_4_)-based multifunctional nanocomposite (PCCN) was used by Zheng et al. to improve hypoxia (Zheng et al., 2016). *In vitro* studies showed that the obtained PCCN could increase the intracellular oxygen concentration and improve the production of reactive oxygen species in hypoxic and normoxic environments. *In vivo* experiments also show that PCCN had good anti-tumor hypoxia ability. Xiong et al. designed a nano platform (IR775@Met@Lip) whose structure is that the liposome is loaded with metformin (MET) and IR775 (Xiong et al., 2021). It can reverse tumor hypoxia, enhance the production of ROS, reduce the expression of PD-L1 and reduce T cell failure. Furthermore, reversing tumor hypoxia successfully inhibited the growth of primary and distal tumors of bladder and colon cancer, respectively.

### Combination Therapy of NP-Based Photothermal Therapy and Photodynamic Therapy Immunotherapy

In the previous discussion, we introduced the research progress of PTT/PDT immunotherapy mediated by different nanomaterials in tumor treatment. The combination of the two methods can not only kill tumor cells through high heat, but also promote the transformation of free radical initiators to toxic free radicals, which further kill tumor cells (Chen et al., 2013). Li et al. designed a nano system composed of ER-targeting pardaxin (FAL) peptides modified-, indocyanine green (ICG) conjugated-hollow gold nanospheres (FAL-ICG-HAuNS) and oxygen-delivering hemoglobin (Hb) liposome (FAL-Hb lipo) to realize PTT and PDT immunotherapy (Li W. et al., 2019). ER-targeted nanosystems induced endoplasmic reticulum stress and calreticulin (CRT) exposure on the cell surface under NIR light irradiation. As a marker of immunogenic cell death (ICD), CRT stimulated the antigen-presenting function of DCs. This activated a series of immune responses, including the proliferation of CD8^+^ T cells and the secretion of cytotoxic cytokines. Chang et al. deposited plasma gold nanoparticles on CMS nanosheets to construct Cu_2_MoS_4_ (CMS)/Au heterostructure (Chang et al., 2020). CMS and CMS/Au can be used as catalase to effectively alleviate tumor hypoxia and enhance the therapeutic effect of O_2_ dependent PDT. It was found that CMS/Au-induced PTT-PDT can induce a strong immune response by promoting dendritic cell maturation, cytokine secretion and activating the response of anti-tumor effector T cells, thereby eliminating primary and metastatic tumors. Yan et al. coated the PDA nanoparticles with UCN and found that in the process of synergy, phototherapy can trigger mature DCs and then activate cytotoxic T lymphocytes cells (CTL) and T memory cells, thus inhibiting tumor metastasis and recurrence. These studies showed that the combination of PTT and PDT can obtain an ideal synergistic effect and start a strong immune response. Liu et al. reported a core shell nanoplatform for enhanced PTT/PDT in the treatment of metastatic breast cancer. The nano system is composed of photosensitizer E6 chloride (Ce6) and rapamycin (RAP) pure drug core and polydopamine (PDA) shell, with the surface is PEGylated (Liu P. et al., 2021). Both *in vitro* and *in vivo* studies showed that coloaded can sensitize PDA based PTT and Ce6 based PDT by inhibiting HSP70 and hypoxia inducible factor-1α (HIF-1α) respectively. In addition, mainly because RAP inhibits matrix metalloproteinases-2 (MMP-2), tumor metastasis is also inhibited.

Although PTT and PDT immunotherapy based on nanoparticles have been widely studied, these studies are still in the laboratory stage and face multiple challenges. For example, there are individual differences in the therapeutic effect of nanomedicines. Furthermore, *in vivo*, the immune responses induced by PTT and PDT are complicated, and their specific manifestations and mechanisms have not been fully understood.

## Nanovaccines for Cancer Therapy

Cancer therapeutic vaccine is a promising class of cancer immunotherapy. However, the effect of cancer vaccines in clinical performance have not been good so far. Nanomaterials provide a unique opportunity to improve the therapeutic effect of cancer vaccines. *In vivo*, nanovaccines have the unique characteristics of improving vaccine efficiency and regulating immune responses (Irvine et al., 2013; Smith et al., 2013). Compared with traditional nano platforms, nanovaccines have some basic advantages. First, coating antigen with nano-carrier can prevent antigen degradation and improve antigen stability. Second, the co-embedding of antigen and adjuvant in nanovaccine can make the antigen and adjuvant co-deliver, thereby enhancing the immunogenicity and therapeutic effect of the vaccine. Furthermore, the multivalent presentation of surface antigens of nanovaccines allow B-cell receptor cross-linking to enhance humoral immune response (Cai et al., 2019). Nanovaccines are usually composed of antigens, molecules or nano adjuvants and/or nano carriers. At present, various nanovaccines based on nanomaterials have been studied, such as those based on cancer neoantigens, mRNA vaccines and biomimetic nanobiomaterials vaccines (Table 1).

**TABLE 1:** Nanovaccines for cancer therapy.

Type	Delivery platform	Immunological effects	References
Neoantigen vaccines	HDL nanodisk	Inducing high levels of antigen-specific CTL responses.	Kuai et al. (2017)
	Acid-activatable micellar nanoparticle	Inducing interferon- β secrete and promoting the activation of T cells and neoantigens.	Zhou et al. (2020b)
	α-melittin-NP	Inducing increased activation of APCs and increased antigen-specific CD8^+^ T cell response.	Yu et al. (2020)
	AC-NPs	Resulting in stronger activation of CD8 + T cells.	Min et al. (2017)
	NPs	Inducing DC maturation, presenting new antigens to CD8^+^ T cells, and enhancing the effect of CD8^+^ CTL activation.	Luo et al. (2021)
mRNA-based vaccines	RNA-LPX	Triggering plasma like DCS and macrophages to release interferon-α (IFN-α).	Kranz et al. (2016)
	LNPs	Activating CD8 T cells after single immunization.	Oberli et al. (2017)
	A2-LNPs	Inducing tumor-infiltrating antigen-specific T cells and IFN-γ strong secretion.	Miao et al. (2019)
	RNA-NPs	Activating high levels of PDL1^+^ CD86^+^ myeloid cells.	Sayour et al. (2018)
	C1 LNP	Inducing the expression of inflammatory cytokines by stimulating the TLR4 signaling pathway of DCs.	Zhang et al. (2021a)
Biomimetic nanobiomaterials-based nanovaccines	CPG-CCNP	Triggering T cell proliferation.	Kroll et al. (2017)
	MOF@FM	Inducing IL-6 and IFN-γ secretion.	Liu et al. (2019b)
	PLGA	Enhancing the uptake and maturation of bone marrow derived DCs.	Xiao et al. (2021)
	PEI modified macrophage membrane	Promoting antigen uptake and proliferation, and finally triggering the effective activation of T cells and strong T cell specific immune response.	Zhang et al. (2020b)
	APC-MS	Promoting the polyclonal expansion of human T cells.	Cheung et al. (2018)
	R-aAPC-IL2	Promoting the proliferation of antigen-specific CD8 + T cells and increasing the secretion of Inflammatory cytokines.	Sun et al. (2017)

### Neoantigen Vaccines

Neoantigens are expressed only in tumor cells and not in any normal cells. So, these new antigens provide an opportunity to use cancer new antigen vaccine to produce a tumor-selective antitumor immune response. Kuai et al. proved that HDL simulated nanodisk binding antigen (Ag) polypeptide and adjuvant can significantly improve the codelivery of Ag/adjuvant to lymphoid organs and maintain the presentation of Ag on DCs (Kuai et al., 2017). Nanodisk-based neoantigen vaccines can induce high levels (∼30%) of antigen-specific CTL responses, especially when combined with immune checkpoint blockade, showing significant tumor therapeutic effects in mouse tumor models. STING pathway is an endogenous mechanism produced by the innate immune system, which can activate and mobilize neoantigen-specific T cells. Because of its key role in tumor immune monitoring, Zhou et al. demonstrated an acid reactive polymeric nanovaccine that activated the STING pathway and improved cancer immunotherapy (Zhou et al., 2020b). Nanovaccines effectively aggregated in lymph nodes, promoted the uptake of DCs and facilitated the release of neoantigens from the cytosol. At the same time, STING agonists activated the STING pathway in DCs and induced interferon-β secretion and promoted the activation of T cells and neoantigens. The study indicated that the development of neoantigen vaccines is particularly useful for enhancing immunity in tumor treatment. Yu et al. demonstrated a self-assembled melittin lipid nanoparticle without additional tumor antigen (α-melittin-NP) can promote the release of tumor antigen *in situ* and lead to the activation of APCs in lymph nodes (LNs) (Yu et al., 2020). Compared with free melittin, α-melittin-NPs significantly increased the activation of APCs, resulting in a 3.6-fold increase in antigen-specific CD8^+^ T cell response. In addition, in the bilateral B16F10 tumor model, α-Melittin-NPs significantly inhibited the growth of primary and distant tumors. Min et al. found that antigen-capturing nanoparticles (AC-NPs) enhanced the presentation of tumor-derived protein antigens (TDPA) by APCs, resulting in stronger activation of CD8^+^ T cells (Min et al., 2017). Luo et al. designed self-assembled polymer NPs with the functions of new antigen capture and self adjuvant. They found that these NPs obtain new antigens from dead cells to form self-adjuvanted molecular activator (SeaMac) nanovaccines, which finally can induce DC maturation, efficiently present new antigens to CD8^+^ T cells, and enhance the effect of CD8^+^ CTL activation (Luo et al., 2017).

### mRNA-Based Vaccines

Combined with the ideal immune stimulation characteristics, mRNA vaccines have outstanding safety and flexibility of gene vaccine. mRNA has been investigated as an attractive vector for the delivery of tumor antigens to DCs. Kranz et al. used liposomes and mRNA to form RNA liposomes (RNA-LPX) complex (Kranz et al., 2016). LPX protected RNA from the influence of extracellular ribonuclease and triggered plasmacytoid DCs and macrophages to release interferon-α (IFN-α). A lipid nanoparticles library (LNPs) was developed by Oberli et al. for the delivery of mRNA vaccines (Oberli et al., 2017). The effectiveness of the vaccine was tested in the invasive B16F10 melanoma model. It was found that CD8^+^ T cells were strongly activated after a single immunization. Miao et al. developed a combinatorial library of ionizable lipid-like materials to facilitate mRNA transmission *in vivo* and to provide an effective and specific immune activation (Miao et al., 2019). These results suggested that in these RNA-loaded ionizable lipid nanovaccines (LNPs), A2-LNPs induced approximately 20-fold stronger secretion of tumor-infiltrating antigen-specific T cells and IFN-γ in a B16F10 mouse melanoma model. Sayour et al. found that the non-targeted tumor RNA encapsulated in lipid NPs are derived from the whole transcriptome and carried excess positive charge, which could initiate the response of peripheral and intratumoral environment to immunotherapy (Sayour et al., 2018). In the immune-resistant tumor model, these RNA NPs activated high levels of PDL1^+^ CD86^+^ myeloid cells, indicating that extensive immune activation of tumor-loaded RNA-NPs, accompanied by inducible PD-L1 expression, could be used for treatment. These studies showed that mRNA nanovaccines have prospective applications in cancer immunotherapy. Zhang et al. developed a minimalist nanovaccine C1 LNP, which can effectively deliver mRNA to APCs, activate toll like receptor 4 (TLR4) and induce strong T cell activation (Zhang H. et al., 2021). In addition, C1 lipid nanoparticle itself induced the expression of inflammatory cytokines such as IL-12 by stimulating the TLR4 signaling pathway of DCs.

### Biomimetic Nanobiomaterials-Based Nanovaccines

As mentioned above, the development of nanotechnology offers a unique approach to facilitate the development of nanovaccines for cancer immunotherapy. To improve the safety and effectiveness of nanovaccines, the use of biomaterials to fabricate multifunctional nanomaterials is emerging (Kang et al., 2018; Li X. et al., 2020).

In recent years, nanocarriers based on cell membrane camouflage have become a biomimetic platform for drug delivery (Guo et al., 2015). Kroll et al. encapsulated CpG adjuvant into poly (lactic-co-glycolic acid) (PLGA) nanoparticles, and then integrated with melanoma cell-derived membrane materials to develop personalized cancer nano vaccine (CpG-CCNP) (Kroll et al., 2017). After vaccination, the T cell proliferation level in the treatment group was the highest. Liu et al. demonstrated a concept of using the biological reprogramming cell membrane of fusion cells (FM) as a tumor vaccine, which was extracted from DCS and cancer cells (Liu W.-L. et al., 2019). In addition, due to the strong passive targeting ability and long cycle profile of metal-organic framework (MOF), they developed a nanovaccine (MOF@FM) using MOF as the carrier of FM. Compared with untreated control group, inoculation MOF@FM can induce increased IL-6 and IFN-γ secretion in experimental groups. Xiao et al. proposed a method for preparing biomimetic cytomembrane nanovaccines (named CCMP@R837) is encapsulated by antigenic cancer cell membrane (CCM)-capped PLGA nanoparticles loaded with imiquimod (R@837) (Xiao et al., 2021). It was found that CCMP@R837 enhanced the uptake and maturation of bone marrow derived DCs and increased the antitumor response to breast cancer 4T1 cells *in vitro*. Zhang et al. have developed a new nano vaccine, which is made by polyethyleneimine (PEI) modified macrophage membrane for co-delivery the antigen (ovalbumin, OVA) and immunostimulant (dendrobium polysaccharides, DP) (Zhang Z. et al., 2020). They found that vaccine can promote antigen uptake and proliferation, and finally trigger the effective activation of T cells and strong T cell specific immune response.

Artificial antigen-presenting cells (aAPCs) are considered as a new bionic nanovaccine which mimics the cellular function of natural APCs to induce tumor-specific immune responses, but does not need to deliver specific antigens and adjuvants required for the activation of natural APCs (Kosmides et al., 2018; Cheng et al., 2020). Cheung et al. described a system simulating natural APCs composed of liquid lipid bilayers supported by mesoporous silica micro rods (Cheung et al., 2018). It was found that APC-simulated scaffolds (APC-MS) could promote the polyclonal expansion of mouse and human T cells. In Kosmides’s group, they found that aAPC can also be directly used for T cell activation *in vivo* during tumor immunotherapy (Kosmides et al., 2017). Sun et al. designed a unique aAPC system based on red blood cell (RBC) by engineering antigen polypeptide loaded main histocompatibility complex -I (MCH- I) and CD28 activated antibody on the surface of RBC, combined with interleukin-2 (IL2) as proliferation and differentiation signal (Sun et al., 2017). The RBC-based aAPC-IL2 (R-aAPC-IL2) can not only provide a flexible cell surface with appropriate biophysical parameters, but also simulate cytokine paracrine. Similar to the function of mature DCs, R-aAPC-IL2 cells can promote the proliferation of antigen-specific CD8^+^ T cells and increase the secretion of inflammatory cytokines to kill tumor cells.

Although many encouraging achievements have been made in the field of cancer nanovaccines in recent years, there are still many challenges to be solved before their clinical transformation and application. As a key step in the development of personalized vaccines, it is still difficult to identify patient specific antigens efficiently and accurately.

## NP-Based T Cell Cancer Immunotherapy

Adoptive cell transfer (ACT) therapy, the infusion of T cells into patients after *in vitro* amplification, has achieved remarkable success in clinical cancer treatment. In particular, chimeric antigen receptor (CAR) T cell therapy, which uses genetically engineered T cells to express tumor antigen recognition receptors, has shown impressive therapeutic effects in patients with hematological malignancies (Neelapu et al., 2017; Brudno and Kochenderfer, 2018; Shah and Fry, 2019). Adoptively transferred T cells recognize cancer cells and kill cancer cells by releasing effective cytotoxic molecules. However, T-cell therapy, including CAR T-cell therapy, has many challenges to be solved in wide application. The key challenges include: 1) Due to insufficient stimulation signals, therapeutic T cells cannot expand *in vivo* to produce a sufficient number of effector cells (Gilham et al., 2012). 2) Due to physical barriers (Caruana et al., 2015) and immunosuppressive environment (Motz et al., 2014), the transport efficiency of T cells to tumor sites is low. 3) Malignant TME leads to therapeutic T cell failure and death (Ren et al., 2017; Bollard et al., 2018). 4) Target gene mutation caused the loss of antigen expression (Hamieh et al., 2019). Among these challenges, the use of nanomaterials may be particularly beneficial to solve the shortage of T cell trafficking and overcome the inhibitory tumor microenvironment.

Schmid et al. designed a PLGA and PEG (PLGA–PEG) nanomaterial modified with antibody for programmed cell death protein 1 (anti-PD-1) to target exhausted T cells (Schmid et al., 2017). This nanomaterial loaded with TGF- β receptor inhibitor compound SD-208 successfully reversed the depletion of T cells *in vivo*. Furthermore, TGF-β delivery of signal inhibitors to PD-1 expressing cells can prolong the survival time of tumor bearing mice. Ou et al. describe the combined delivery of tLyp1 peptide modified regulatory T (Treg) cell-targeted hybrid NPs (tLyp1-hNPs) for targeting neuropilin-1 (Nrp1) receptors on Treg cells and well-known anti CTLA4 immune checkpoint inhibitors (Ou et al., 2018). The Treg cell inhibitory drug IMT was encapsulated in tLyp1-hNPs. *In vivo* studies have shown that the synergistic antibody of tLyp1-hNPs loaded with IMT and anti CTLA4 can activate the strong immune response against tumor by down regulating immunosuppressive Treg cells and activating CD8^+^ T cells. Huang et al. reported a dual-mechanism based cytotoxic T cells (CTL) infiltration enhancer Nano-sapper, which can simultaneously reduce physical obstacles in TME and recruit CTL to enhance immunotherapy of immune-excluded tumor (IET) (Huang et al., 2020). Nano-sapper can reverse the abnormally activated fibroblasts, reduce collagen deposition, normalize the blood vessels in the tumor, and stimulate the expression of chemical attractants of recruited lymphocytes *in situ*, so as to reshape the TME.

Although T-cell cancer immunotherapy based on nanomaterials has made great progress in recent years, how to control the behavior of adoptive transfer T cells *in vivo* needs to be solved urgently.

## NP-Based Bacteria Cancer Immunotherapy

Bacteria based tumor immunotherapy has attracted academic attention because of its unique mechanism and rich application in triggering host anti-tumor immunity. Furthermore, it is considered that the integration of bacteria and nanomaterials for multifunctional synergistic therapy is a promising treatment (Toussaint et al., 2013). Due to the different synthetic methods and encapsulated drugs, nanomaterials can achieve a variety of functions in cancer treatment (Xu et al., 2020; Yang et al., 2020). Therefore, it seems more direct to integrate nanomaterials and other functions on the outer membrane of bacteria.

Liu et al. integrated photosensitizer-encapsulated nanoparticles on the bacterial surface through amide bonds to engineer photosynthetic bacteria (Synechococcus 7942, Syne) (Liu et al., 2020). Under 660 nm laser irradiation, Syne can continuously produce oxygen through photosynthesis, which greatly improves tumor hypoxia and produces more ROS. PDT promoted by photosynthesis not only can inhibit the growth of primary tumors, but also can reverse the TME of immune suppression to immune response, and prevent tumor recurrence even in the triple-negative breast cancer (TNBC) mouse model. Li et al. designed pathogen mimicking nano-pathogenoids (NPNs) containing pathogen-associated molecular patterns (PAMPs) through cloaking NPs with outer-membrane vesicles (OMVs), which can be recognized by pattern recognition receptors (PRRs) on neutrophils (Li M. et al., 2020). Neutrophils move to the inflamed tumor, exude through blood vessels and penetrate the tumor. After inflammatory stimulation, neutrophils rapidly release NPN, which is then absorbed by tumor cells to exert anticancer effects. Chen et al. designed a eukaryotic–prokaryotic vesicle (EPV) nanoplatform by fusing melanoma cytomembrane vesicles (CMVs) and attenuated Salmonella OMVs (Chen et al., 2020). *In vivo* prophylactic trials have shown that EPV nanoparticles can act as a prophylactic vaccine, stimulating the immune system and triggering an anti-tumor immune response against tumorigenesis.

The biggest advantage of integrating nanomaterials on the surface of bacterial outer membrane is easy preparation and low cost, but there are also some shortcomings. First of all, if a dense and continuous shell is formed when nanomaterials are integrated on the outer membrane of bacteria, the bacteria will be completely surrounded, so as to weaken the targeting ability of bacteria. Furthermore, appropriate functions should be selected in combination with the bacterial body to produce synergistic rather than antagonistic effects.

## Conclusion and Outlook

Over the past decades, cancer immunotherapy has achieved tremendous clinical success and become a potential mainstay therapy for cancer treatment. However, due to the low responsiveness of tumor antigens and numerous escape mechanisms in the immunosuppressive microenvironment, the clinical application of immunotherapy is frequently accompanied by low response rates, short durations, and the induction of severe immunotoxicity. The emergence of nanomedicine offers a new way of thinking for cancer immunotherapy, and plays an important role in modulating tumor immune responses. Nanomaterials can be used to modulate the tumor suppressive microenvironment by targeting immunosuppressive cells, immunosuppressive factors or effector T cells. Nanomaterials usually involve in the following roles: 1) activating cellular immunity by enhancing tumor immunogenicity and promoting antigen presentation, and 2) promoting the activation of tumor-infiltrating NK cells to achieve local activation of the immune system. NP-based PTT and PDT can lead to a large number of tumor cell deaths and trigger immune responses, including redistribution and activation of immune effector cells, expression and secretion of cytokines, and transformation of memory T lymphocytes. Furthermore, the combination of two methods can obtain ideal synergistic effect and start a strong immune response. But *in vivo*, the immune responses induced by PTT and PDT are complex, and the specific performance and mechanisms are not fully understood. Nanovaccine is another application based on NPs, which recognizes and attacks tumor cells by activating adaptive immune system, thereby triggering anti-tumor immune response. Compared with traditional nano platforms, nanovaccines have some basic advantages, such as coating antigen with nano-carrier to prevent antigen degradation, co-embedding of antigen and adjuvant to enhance the immunogenicity and therapeutic effect of the vaccine. However, there are still many challenges that need to be solved before their clinical transformation and application. As a key step of personalized vaccine development, it is still difficult to identify patient specific antigens efficiently and accurately. T-cell cancer immunotherapy based on nanomaterials has made great progress in recent years. However, how to control the behavior of adoptive transfer T cells *in vivo* needs to be solved urgently in the future. Integrating nanomaterials on the surface of bacterial outer membrane is easy preparation and low cost, but there are also some shortcomings that need to be overcome.

In summary, most nanomaterials for cancer immunotherapy are still in the experimental stage and are still far from clinical application on account of safety, reproducibility, and individual differences. In order to achieve the clinical applications of nanomaterials, it is essential to further develop nanoparticles. First, optimize the carrier materials to achieve good biodegradable and biocompatibility to provide safe drugs for clinical patients; then optimize the production process of nanomedicines to achieve high-volume production and stable quality control to provide stable drug pathways for clinical patients; third, optimize the different ratios of various drugs in combination to achieve the best therapeutic effect; optimize the targeting effect of drugs to achieve precise control of drug release and clarify the timing of activating immunotherapy. It is believed that with the development of nanotechnology and the continuous research on the mechanism of tumor immunotherapy, nanomaterials will play a powerful role in promoting tumor immunotherapy in clinical.
